# Design and Validation of the Gender-Based Violence Stereotypical Beliefs Scale

**DOI:** 10.3390/bs14111093

**Published:** 2024-11-14

**Authors:** Enrique Bonilla-Algovia, Andreea Gabriela Pana, Concepción Carrasco Carpio

**Affiliations:** 1 Department of Education, Distance University of Madrid (UDIMA), 28400 Madrid, Spain; 2 Department of Economics (Sociology), University of Alcalá (UAH), 28801 Madrid, Spain; andrea.pana@uah.es (A.G.P.); concha.carrasco@uah.es (C.C.C.)

**Keywords:** gender-based violence, violence against women, myths about violence, validation

## Abstract

Gender-based violence is a public health issue influenced by culture and social values, which is why its comprehensive prevention requires addressing distorted beliefs and legitimising myths present in society. The aim of this study was, on the one hand, to design and validate the Gender-Based Violence Stereotypical Beliefs Scale (GBVSBS), and, on the other, to analyse the differences between men and women regarding these beliefs. The sample consisted of 404 university students from the Community of Madrid and Castilla-La Mancha (Spain), aged between 18 and 53 years. This study is based on a quantitative methodology and a cross-sectional design. The judgement of four specialists in the field supported the content validity of the items. Factorial analyses provided evidence for a two-factor model: myths about male perpetrators and myths about gender-based violence and female victims. The fit indices and reliability coefficients were adequate. Stereotypical beliefs about gender-based violence correlated with victim-blaming attitudes, and different levels of acceptance were found depending on gender. In conclusion, this study offers a valid and reliable instrument with which to analyse the sociocultural beliefs surrounding gender-based violence today, promoting the implementation of socio-educational interventions.

## 1. Introduction

Currently, violence against women is not only a public health issue that violates human rights globally, but also a barrier to countries and their diverse societies advancing towards sustainable development. Approximately one in three women worldwide has been a victim of intimate partner violence or non-partner sexual violence [1]. This form of violence, also known as gender-based violence, has various consequences for the physical and mental health of women and their children, as well as social, occupational, and economic repercussions [2,3,4,5,6,7,8,9,10,11,12,13,14].

Studies have detected gender-based violence in every country where research has been conducted. The European Union Agency for Fundamental Rights survey [15] shows that 22% of ever-partnered women have suffered physical and/or sexual violence by an intimate partner; 22% of women have experienced physical and/or sexual violence by someone other than a partner; and 33% have suffered violence from a partner, a non-partner, or both. In Spain specifically, 13% of women have been subjected to physical and/or sexual violence by a partner, and 16% by someone other than a partner. The prevalence rates of gender-based violence vary across different countries, suggesting that this form of violence can be eradicated through public policies and prevention and awareness programmes [16].

The European Commission’s Gender Equality Strategy 2020–2025 outlines the commitments and courses of action of the Member States regarding gender equality. Similarly to the 2030 Agenda for Sustainable Development, one of the objectives of the Gender Equality Strategy is the eradication of gender-based violence [17] In line with this strategy, as highlighted in the 2024 Report on Gender Equality, the European Union in 2023 adhered to the Council of Europe Convention on Preventing and Combating Violence against Women and Domestic Violence, more commonly known as the Istanbul Convention [18]. The initial results of the EU Survey on Gender-Based Violence against Women and Other Forms of Interpersonal Violence (EU-GBV) did not include prevalence data for Spain [19]. However, pending the final report, preliminary data from [20] indicate that 20% of women residing in Spain (aged 15 and over) have experienced physical or sexual violence by a non-partner, and 14.4% of those who have ever had a partner have suffered physical or sexual violence by an intimate partner.

Gender-based violence, far from being an individual issue, is shaped by culture and social values. Society’s perception of gender-based violence is formed by beliefs that seek to explain its causes and consequences, and it can be made up of both erroneous and irrational beliefs as well as beliefs grounded in empirical evidence. The former have become the focus of scientific study due to their impact on society at large and women specifically. Addressing these beliefs allows for a comprehensive analysis of the problem [21]. In this regard, these myths or distorted beliefs about gender-based violence are based on biases and falsehoods that not only hinder its understanding and interpretation, but also its eradication and prevention [22,23]. Myths are present in both real and virtual environments, affecting the understanding of various forms of gender-based violence, such as intimate partner violence [24,25], sexual violence [26,27], and even cybersexual violence [28].

Myths or stereotypical beliefs about gender-based violence fall into various groups, such as the minimisation of abuse (e.g., gender-based violence is a one-off problem), the exoneration of men who perpetrate it (e.g., men who commit gender-based violence have mental health issues), the blaming of female victims (e.g., women could leave the abusive relationship if they really wanted to), the reduction in violence to individual factors (e.g., gender-based violence occurs in families with financial problems), the denial of gender-based violence (e.g., most reports of gender-based violence are false), or the neo-myths that portray men as victims of the system (e.g., men are the real victims of the system) [23]. Therefore, it should be noted that all myths impact both the perpetration of gender-based violence and the social responses to it, encompassing the reactions of institutions, other individuals, and the victims themselves [25,29,30].

The academic literature shows that beliefs about the different forms of gender-based violence are related to gender socialisation. Previous studies have evidenced that men, compared to women, tend to support more myths about sexual aggression [26,27]; myths about intimate partner violence against women [31,32]; blaming attitudes towards women victims of intimate partner violence [25] biases regarding women’s inferiority, the justification of violence as an acceptable way to solve problems, victim blaming, the minimization of violence, and the exoneration of aggressors [24,33,34]; and explicit and implicit attitudes about intimate partner violence against women [35]. Consequently, gender differences provide additional validity evidence for any scale measuring myths about gender-based violence.

Social perceptions surrounding gender-based violence evolve alongside cultures and societies, meaning that myths become more implicit. This highlights the need for tools assessing sociocultural issues to be constantly updated to avoid becoming outdated. Moreover, the scales developed to date for evaluating beliefs about gender-based violence have primarily focused on one of the two most common forms of violence: intimate partner violence [24,25,31] or sexual violence [26,27]. In this regard, these scales tend to focus specifically on one type of violence rather than addressing both simultaneously. To overcome these limitations, the Gender-Based Violence Stereotypical Beliefs Scale (GBVSBS) was designed, an updated tool that measures both myths about intimate partner violence and myths about sexual violence, both within and outside of relationships. The innovation of the GBVSBS lies in its alignment with the most current conceptualizations of gender-based violence, as it incorporates a comprehensive perspective that encompasses the two most widespread forms of violence against women. Consequently, the present research aims to design and validate the GBVSBS in a sample of university students, as well as to analyse gender differences in beliefs about violence.

## 2. Method

### 2.1. Participants

The study involved 404 university students (83.42% women and 16.09% men) from the Community of Madrid and Castilla-La Mancha (Spain). Data collection was carried out through intentional, non-random sampling among students enrolled in undergraduate and master’s programmes. The sociodemographic characteristics of the sample can be found in Table 1. Most participants were Spanish nationals (98.27%). The average age was 21.36 years (SD = 4.37), although ages ranged from 18 to 53 years. The majority of students were in their first year (46.78%), although there were students from various academic years. A total of 90.84% were pursuing an undergraduate degree, while 9.16% were enrolled in a master’s programme. The proportions of individuals with and without a partner were very similar.

### 2.2. Instrument

1. Sociodemographic characteristics: sex, age, nationality, year of study, relationship status, etc.

2. Gender-Based Violence Stereotypical Beliefs Scale (GBVSBS). This instrument has been designed to examine myths or stereotypical beliefs about gender-based violence. It is an integrative scale that allows for the simultaneous analysis of myths surrounding intimate partner violence and myths related to sexual violence (both within and outside of a relationship). It captures myths that exonerate male aggressors (e.g., “Men who perpetrate gender-based violence have mental health problems”), myths that blame female victims (e.g., “Many women suffer gender-based violence because they push men to their limits” and “Women are sexually assaulted because they drink too much and are careless”), myths that reduce the problem to certain groups or extraordinary factors (e.g., “Gender-based violence only occurs in countries with low levels of development”), and myths that minimise intimate partner violence and sexual violence (e.g., “Gender-based violence does not exist or only occurs in isolated cases”). It also addresses neo-myths (e.g., “Most women who report having been sexually assaulted are seeking legal benefits”). It consists of 26 items, whose wording and suitability have been evaluated by a group of experts in gender studies. The response format is a 5-point Likert scale: 1, strongly disagree; 2, disagree; 3, neither agree nor disagree; 4, agree; and 5, strongly agree. All items are phrased in a direct manner, so higher scores indicate more stereotypical beliefs. The Cronbach’s alpha obtained in the present study was 0.91.

3. Victim-Blaming Attitudes in Cases of Intimate Partner Violence against Women Scale (VB-IPVAW; 25). The VB-IPVAW measures various attitudes that blame women victims of gender-based violence in the context of intimate relationships. The validation of the scale was conducted with a sample of 1800 participants from Spain, aged between 18 and 75 years. The scale is unidimensional, consisting of 12 items (e.g., “Men are violent towards their partners because they make them jealous”) that are grouped into a single dimension. The response format is a 4-point Likert scale: from 1, strongly disagree, to 4, strongly agree. The Cronbach’s alpha obtained during validation was adequate (α > 0.70). Similarly, the alpha coefficient obtained in the present research was 0.82.

### 2.3. Procedure

The research team developed the structured questionnaire and selected the participant sample based on the study’s objectives. Participation was voluntary, and the information was collected anonymously. Prior to data collection, the Participant Information Sheet (PIS) and Informed Consent (IC) form were provided, and participants gave their consent after being informed about the study’s details. Responses were recorded via an online survey using Microsoft Forms. Completing the questionnaire took approximately 30–40 min. This study was approved by the Research Ethics and Animal Experimentation Committee of the University of Alcalá (ethics code: CEI: CEIP/2022/06/102 and CEI: CEID/2022/3/058). The work is part of a research project funded by grants from the Department of Education, Culture and Sports (Castilla-La Mancha) for the implementation of scientific research and technology transfer projects in 2021, co-financed by the European Regional Development Fund (ERDF).

### 2.4. Analyses

Statistical analyses were conducted using both SPSS statistical software (IBM 24.0) and the FACTOR Analysis statistical programme (14.1.0.0). Firstly, the content validity of the GBVSBS was evaluated by using the expert judgement technique. Secondly, to obtain the descriptive statistics of the GBVSBS items and to analyse their psychometric properties, the mean (with 95% confidence intervals), standard deviation, standard error, skewness, kurtosis, corrected item–total correlation, and Cronbach’s alpha if the item was deleted were examined. Thirdly, the factor structure of the scale was explored through a parallel analysis (PA), one of the most recommended techniques at present [36]. Since the variables are ordinal and the data do not meet the assumption of normality, PA was performed on polychoric correlations by using the robust unweighted least squares (ULS) method. Additionally, as complementary validity analyses, the following goodness-of-fit tests were assessed [37]: χ^2^/df, GFI, AGFI, CFI, NNFI, RMSEA, and RMSR. Fourthly, Cronbach’s alpha, Omega, and ordinal alpha for the GBVSBS were analysed. To complete the validation process, Pearson correlations between the GBVSBS and the VB-IPVAW [25] were examined to verify convergent validity. Finally, once validity evidence was obtained, gender differences in myths about gender-based violence from the GBVSBS were explored.

## 3. Results

### 3.1. Content Validity of the GBVSBS

The list of GBVSBS items was sent to four specialists in gender studies, with the aim of having them conduct a quantitative and qualitative evaluation. The quantitative evaluation involved a five-point Likert scale that assessed clarity (1, not clear at all; 5, very clear), relevance (1, not relevant at all; 5, very relevant), and appropriateness (1, not appropriate at all; 5, very appropriate) of the items. The qualitative evaluation consisted of an open-ended question for providing observations. Following the review of both types of evaluations, the 26 items included in Table 2 were incorporated into the scale. The mean item scores for clarity (M = 4.96; SD = 0.14), relevance (M = 4.98; SD = 0.09), and appropriateness (M = 4.96; SD = 0.14) were high. The list of GBVSBS items was sent to 4 specialists in Gender Studies for quantitative and qualitative evaluation.

### 3.2. Descriptive Analyses of the GBVSBS Items

The mean score for the GBVSBS items was 37.44 (see Table 3). The lowest score was found for item 15 (*Gender-based violence is a problem of the past that has already been overcome*), while the highest score was for item 5 (*Men who perpetrate gender-based violence have mental health problems*). The standard deviation was less than or close to one for all items, and the standard errors were low. Furthermore, Cronbach’s alpha was 0.913 (α = 95% CI = 0.90–0.93), and the lowest corrected item–total correlation was 0.37. Removing any item would reduce the reliability of the scale, except for item 5, which would slightly improve it; thus, all items were retained. As indicated by the kurtosis values (ranging from −1.28 to 33.21) and skewness values (ranging from 0.24 to 5.13), a normal distribution of the data cannot be assumed; therefore, the factor structure analysis was conducted on polychoric correlations by using the ULS method.

#### Dimensional Analysis of the Scale

The Kaiser–Meyer–Olkin (KMO) test and Bartlett’s statistic yielded adequate values (KMO = 0.90; Bartlett’s χ^2^ = 5670.75, *p* < 0.001), supporting the suitability of the data for conducting factor analysis. Considering these results, a parallel analysis was performed to study the factor structure of the GBVSBS, using the robust ULS method. First, the goodness-of-fit of the one-factor model that grouped the 26 items was assessed. The results indicated an acceptable fit (χ^2^/df = 4.86; GFI = 0.96; AGFI = 0.96; CFI = 0.98; NNFI = 0.98; RMSEA = 0.08; and RMSR = 0.10). However, the FACTOR programme suggested that a two-factor model might be more appropriate for the GBVSBS. Consequently, the goodness-of-fit of the new model was analysed, resulting in a better fit across all indices (χ^2^/df = 1.83; GFI = 0.99; AGFI = 0.99; CFI = 0.99; NNFI = 0.99; RMSEA = 0.04; and RMSR = 0.06). This model groups the items into two dimensions: myths about aggressive men (items 5, 6, 7, 8, and 9) and myths about gender-based violence and female victims (the remaining items). The reliability values obtained for the GBVSBS are adequate, with values exceeding 0.70 for the ordinal alpha, Omega, and Cronbach’s alpha. The factor loadings were above 0.35 in the items of both factor models (see Table 4). In the one-factor model, the factor weights ranged from 0.40 to 0.84; in the two-factor model, the weights ranged from 0.43 to 0.91 in the first dimension and from 0.46 to 0.94 in the second dimension. Therefore, in line with the findings from the previous analyses, all items of the GBVSBS exhibit good psychometric properties.

### 3.3. Convergent Validity of the GBVSBS

The convergent validity of the GBVSBS was analysed through Pearson correlations between it and the VB-IPVAW [25], which assesses victim-blaming attitudes towards women in cases of gender-based violence. The results indicated that the correlations between the two scales are strong and direct (*r* = 0.68, *p* < 0.001), suggesting that they assess similar constructs. The VB-IPVAW shows a higher correlation with myths about gender-based violence and female victims (*r* = 0.68, *p* < 0.001) than with myths about aggressive men (*r* = 0.39, *p* < 0.001).

### 3.4. Differences Between Men and Women in the GBVSBS

The mean scores obtained on the GBVSBS vary by sex. Men, compared to women, exhibit higher levels of acceptance of myths about gender-based violence, with statistically significant differences (M_men_ = 1.67, SD = 0.47; M_women_ = 1.40; SD = 0.42; *t* = 4.61, *p* < 0.001). Regarding the dimensions of the GBVSBS, although both sexes score higher on the myths about aggressive men (M_men_ = 2.05, SD = 0.76; M_women_ = 1.82; SD = 0.78) than on the myths about gender-based violence and female victims (M_men_ = 1.58, SD = 0.48; M_women_ = 1.30; SD = 0.41), men demonstrate more distorted beliefs than women for both factors: factor 1 (*t* = 4.38, *p* < 0.001) and factor 2 (*t* = 2.17, *p* < 0.05).

## 4. Discussion

Most countries have initiated actions and policies against gender-based violence, as it is a social problem in the European Union and worldwide [1,14,15,17,18,19,20], with multifactorial consequences for both women and society as a whole [2,3,4,5,6,7,8,9,10,11,12,13,14]. 

Spain is no exception, despite the feminist advances of recent decades. The levels of intimate partner violence and sexual violence remain concerning [15,20] and not only demand the implementation of preventive actions at all levels but also require reflection on the sociocultural values that underpin gender inequality and the various forms of violence against women. These values, composed of myths or stereotypical beliefs, distort the causes of the problem and serve to minimise and justify it [21,23], affecting prevention and social, community, and individual responses to violence [25,29,30].

The scientific approach to attitudes and stereotypical beliefs about gender-based violence has grown in recent years as social imaginaries evolve and adapt to the conditions of each historical moment. This necessitates that scales assessing social issues are continuously reviewed and updated. Furthermore, given the specificity of previous scales regarding beliefs or attitudes towards gender-based violence [24,25,26,27,31], there is a need for reliable scales that simultaneously assess myths surrounding intimate partner violence and myths related to sexual violence (both within and outside of relationships). By integrating two of the most common forms of gender-based violence into the same instrument, it provides a comprehensive perspective that aligns with new conceptualizations, expanding the conceptual boundaries of gender-based violence rather than restricting it to the realm of intimate partnerships. Consequently, this research has involved the design and validation of the GBVSBS, an updated scale that enables a comprehensive analysis of stereotypical beliefs about both forms of gender-based violence.

A group of specialists in gender studies conducted an expert review to determine whether the items adequately represented the stereotypical beliefs about the issue at hand [37], achieving high scores across all domains, which certifies the content validity of the GBVSBS items. On the other hand, preliminary analyses show that all items are important for the overall scale, as the Cronbach’s alpha did not increase with the removal of any elements, and the corrected item–total correlation was above 0.30 in all cases. The only item whose removal improved reliability was item 5 (Men who perpetrate gender-based violence have mental health problems), but the change in Cronbach’s alpha was not significant (α-item = 0.005). Therefore, it can be concluded that the 26 items of the GBVSBS are clear, relevant, pertinent, and adequately represent the construct under analysis, namely, the stereotypical beliefs about gender-based violence.

The factor analyses revealed that the two-factor model provided better fit indices compared to the unifactorial model [37]. Thus, the stereotypical beliefs in the GBVSBS are primarily grouped into two dimensions: myths about aggressive men and myths about gender-based violence and victimised women. The first dimension contains beliefs that focus attention on specific factors that exonerate aggressive men (e.g., alcohol, drugs, work-related issues, etc.), while the second dimension includes beliefs that place responsibility on the women victims, while simultaneously minimising, denying, and justifying gender-based violence (both intimate partner violence and sexual violence within and outside of relationships). Furthermore, this second dimension encompasses denial narratives that argue that gender-based violence is a construct of feminism intended to harm men and benefit women [23].

Attitudes of victim blaming towards women in cases of gender-based violence [25] were found to correlate with the stereotypical beliefs from the GBVSBS. The obtained relationships were positive and significant for both myths about aggressive men and myths about gender-based violence and victimised women. These findings not only verify convergent validity but also confirm the suitability of the GBVSBS for analysing discourses that blame women victims.

Previous research at both national and international levels has found that men, compared to women, tend to hold more distorted beliefs about the causes and consequences of gender-based violence, whether in myths about sexual aggression or in other myths concerning intimate partner violence against women [24,25,26,27,31,32,33,34,35]. In the same vein, the results of this study show that men have a more biassed perspective on gender-based violence compared to women, indicating that prevention efforts must consider the role of gender socialisation in perpetuating inequality and violence.

The research is not without limitations that warrant specific consideration before drawing conclusions from the obtained results. The number of participants is sufficient to analyse the psychometric properties of the scale [37], but it is not a representative sample of the young population in Spain. Additionally, there is a disproportion between the number of women and men in the sample, which is due to the fact that the participants are studying undergraduate and master’s degrees. Furthermore, although honesty was requested and confidentiality of the data was assured, the responses may be affected by social desirability. To mitigate this, an information sheet was provided that included a commitment to data anonymisation. Lastly, considering that social perceptions surrounding gender-based violence depend on context, potential variations in the factor structure should be examined in other cultural contexts different from Spain (e.g., Latin America). In the future, given the structural nature of gender inequality, it would be beneficial to analyse stereotypical beliefs about gender-based violence based on other variables (e.g., educational level, training, religiosity, political ideology, and age) and to study their relationship with other constructs (e.g., sexism and attitudes toward equality).

In conclusion, the present research provides a valid and reliable instrument for analysing myths or stereotypical beliefs about gender-based violence, incorporating beliefs that justify and minimise both intimate partner violence and sexual violence against women. The GBVSBS has not only been designed for research purposes but can also guide the implementation of educational interventions and preventive actions against gender-based violence. Combating the cultural values, social norms, and individual beliefs that sustain violence is one of the most effective strategies for its prevention [16].

## Figures and Tables

**Table 1:** Sociodemographic characteristics of the sample.

	n	%
Sex		
Men	65	16.09%
Women	337	83.42%
Age		
18–20 years	214	52.97%
21–24 years	158	39.11%
25 years and older	32	7.92%
Nationality		
Spanish	397	98.27%
Foreign	7	1.73%
Educational Level		
Undergraduate	367	90.84%
Master’s	37	9.16%
Relationship Status		
With partner	201	49.75%
Without partner	203	50.25%

**Table 2:** Formulation of the items and averages of the specialist judgements.

	M (Clarity)	M (Relevance)	M (Appropriateness)
1. Gender-based violence only occurs in countries with low levels of development.	5	5	5
2. Most gender-based violence occurs in dysfunctional families or in socially vulnerable contexts.	5	5	5
3. Gender-based violence is a problem imported from other cultures and countries.	5	5	5
4. Gender-based violence is primarily caused by men of foreign origin.	5	5	5
5. Men who perpetrate gender-based violence have mental health problems.	5	5	5
6. Men commit gender-based violence because they consume alcohol or drugs.	5	5	5
7. Men commit gender-based violence because of gambling or betting addiction.	5	5	5
8. Men commit gender-based violence because they have work-related concerns.	5	5	5
9. Many men commit gender-based violence because women make them lose their temper.	4.5	5	5
10. Abused women who tolerate gender-based violence are also partly to blame.	5	5	5
11. Some women suffer gender-based violence because they provoke men.	5	5	5
12. Many women suffer gender-based violence because they push men to their limits.	5	5	5
13. Gender-based violence is a feminist invention.	5	5	4.5
14. Gender-based violence does not exist or exists only in isolated cases.	4.5	4.5	4.5
15. Gender-based violence is a problem of the past that has already been overcome.	5	5	5
16. A significant number of reports of gender-based violence are unfounded.	5	5	5
17. Women use the issue of gender-based violence for their own benefit.	5	5	5
18. Laws against gender-based violence should no longer exist today.	5	5	5
19. Nowadays, anything can be interpreted as an act of gender-based violence.	5	5	5
20. Gender-based violence is exaggerated in today’s society.	5	5	5
21. Gender-based violence should be resolved in the private sphere.	5	5	5
22. Many women suffer sexual violence because they dress provocatively.	5	5	5
23. Women are sexually assaulted because they drink too much and are careless.	5	5	5
24. If a woman does not resist, it is not considered rape.	5	5	5
25. Many women flirt with men, but then complain that these men sexually assault them.	5	5	5
26. Most women who report having been sexually assaulted are seeking legal benefits.	5	5	5

Note: M = mean.

**Table 3:** Descriptive statistics of the items of the GBVSBS.

	M	CI 95%	SD	SE	Kurtosis	Skewness	*r* *_item–total_* ^c^	α-Item
Item 1	1.17	1.12–1.23	0.54	0.03	16.10	3.79	1.27	0.912
Item 2	1.86	1.76–1.97	1.09	0.05	0.09	1.06	0.50	0.911
Item 3	1.40	1.32–1.48	0.78	0.04	4.90	2.20	0.47	0.910
Item 4	1.49	1.41–1.57	0.83	0.04	2.38	1.72	0.57	0.908
Item 5	2.67	2.53–2.81	1.44	0.07	−1.28	0.24	0.37	0.918
Item 6	1.92	1.82–2.02	1.02	0.05	−0.28	0.83	0.54	0.909
Item 7	1.77	1.67–1.86	0.95	0.05	−0.05	1.00	0.53	0.910
Item 8	1.57	1.48–1.65	0.86	0.04	0.68	1.34	0.53	0.909
Item 9	1.37	1.30–1.45	0.77	0.04	5.42	2.30	0.51	0.910
Item 10	1.46	1.37–1.54	0.91	0.05	3.59	2.08	0.55	0.909
Item 11	1.20	1.14–1.25	0.53	0.03	7.84	2.85	0.60	0.909
Item 12	1.17	1.12–1.21	0.46	0.02	8.95	2.97	0.61	0.909
Item 13	1.12	1.08–1.17	0.44	0.02	16.32	3.95	0.41	0.912
Item 14	1.10	1.06–1.14	0.40	0.02	33.21	5.13	0.45	0.911
Item 15	1.08	1.05–1.11	0.32	0.02	26.75	4.72	0.47	0.912
Item 16	1.50	1.42–1.58	0.80	0.04	2.10	1.62	0.58	0.908
Item 17	1.57	1.49–1.65	0.82	0.04	1.62	1.43	0.67	0.907
Item 18	1.18	1.12–1.24	0.60	0.03	17.68	4.03	0.37	0.912
Item 19	1.64	1.55–1.73	0.96	0.05	1.40	1.47	0.72	0.905
Item 20	1.54	1.45–1.63	0.92	0.05	2.00	1.67	0.69	0.906
Item 21	1.27	1.22–1.33	0.60	0.03	5.62	2.39	0.48	0.910
Item 22	1.31	1.23–1.39	0.80	0.04	8.06	2.88	0.57	0.909
Item 23	1.18	1.13–1.24	0.54	0.03	12.88	3.40	0.57	0.909
Item 24	1.16	1.11–1.21	0.55	0.03	20.13	4.23	0.47	0.911
Item 25	1.36	1.28–1.44	0.78	0.04	5.41	2.38	0.68	0.907
Item 26	1.37	1.30–1.44	0.72	0.04	4.51	2.11	0.67	0.907

Note: N = 404; M = media; CI 95% = confidence interval of the mean; SD = standard deviation; SE = standard error; *r_item–total_*^c^ = corrected total correlation; and α = Cronbach’s alpha if the element is deleted.

**Table 4:** Factor weights of the items and reliability of the models.

	One-Factor Model	Two-Factor Model	
		Factor 1	Factor 2
Item 1	0.62	0.64	
Item 2	0.58	0.44	
Item 3	0.62	0.43	
Item 4	0.70	0.61	
Item 5	0.40		0.46
Item 6	0.55		0.92
Item 7	0.54		0.94
Item 8	0.59		0.85
Item 9	0.68		0.47
Item 10	0.71	0.61	
Item 11	0.82	0.70	
Item 12	0.83	0.67	
Item 13	0.69	0.87	
Item 14	0.74	0.91	
Item 15	0.79	0.91	
Item 16	0.73	0.72	
Item 17	0.80	0.78	
Item 18	0.64	0.76	
Item 19	0.84	0.84	
Item 20	0.83	0.84	
Item 21	0.67	0.53	
Item 22	0.79	0.72	
Item 23	0.79	0.68	
Item 24	0.75	0.75	
Item 25	0.84	0.76	
Item 26	0.82	0.76	
Cronbach´s alpha	0.91	0.91	0.81
Ordinal alpha	0.96	0.96	0.84
Omega	0.96	0.96	0.86

## Data Availability

Data is contained within the article.
